# Animal Models, Prophylaxis, and Therapeutics for Arenavirus Infections

**DOI:** 10.3390/v4091802

**Published:** 2012-09-24

**Authors:** Eric Vela

**Affiliations:** 1 Battelle, 505 King Ave, Columbus, OH 43201, USA; 2 Center for Predictive Medicine, University of Louisville, 950 N. Hurstbourne Parkway, Louisville, KY 40222, USA; Email: velae@battelle.org; Tel.: +1-614-424-7998 ; Fax: +1-614-458-7998.

**Keywords:** *Arenavirus*, *Prophylaxis*, *Therapeutics*, Hemorrhagic Fever, Animal Models

## Abstract

Arenaviruses are enveloped, bipartite negative single-stranded RNA viruses that can cause a wide spectrum of disease in humans and experimental animals including hemorrhagic fever. The majority of these viruses are rodent-borne and the arenavirus family can be divided into two groups: the Lassa-Lymphocytic choriomeningitis serocomplex and the Tacaribe serocomplex. Arenavirus-induced disease may include characteristic symptoms ranging from fever, malaise, body aches, petechiae, dehydration, hemorrhage, organ failure, shock, and in severe cases death. Currently, there are few prophylactic and therapeutic treatments available for arenavirus-induced symptoms. Supportive care and ribavirin remain the predominant strategies for treating most of the arenavirus-induced diseases. Therefore, efficacy testing of novel therapeutic and prophylactic strategies in relevant animal models is necessary. Because of the potential for person-to-person spread, the ability to cause lethal or debilitating disease in humans, limited treatment options, and potential as a bio-weapon, the development of prophylactics and therapeutics is essential. This article reviews the current arenavirus animal models and prophylactic and therapeutic strategies under development to treat arenavirus infection.

## 1. Arenavirus Introduction

Viral hemorrhagic fever (VHF) syndrome has the potential to occur in individuals infected with a virus from one of the four RNA virus families, *Filoviridae, Arenaviridae, Bunyaviridae, *and *Flaviviridae*. Arenaviruses are a diverse group of negative-stranded, bi-partite RNA viruses that can potentially cause hemorrhagic fever in animals and humans. Several arenaviruses have been confirmed to cause disease in humans including Lymphocytic choriomeningitis virus (LCMV), Lujo virus (LUJV), Lassa virus (LASV), Guanarito virus (GTOV), Junín virus (JUNV), Machupo virus (MACV), Sabia virus (SABV) and Chapare virus. VHF produces a wide range of symptoms and pathology within infected individuals, which is typically dependant on the specific causative virus, but some similar disease symptoms do occur. For instance, infected individuals typically present with fever, malaise, body aches, petechial rash, dehydration, and internal and external hemorrhage. Advanced stages of VHF may result in mortality. 

Rodents are the principal reservoirs for most of the arenaviruses in nature, and all of the arenaviruses that cause disease are rodent-borne [1]. Bats (*Artibeus spp*) are presumably the natural reservoir for Tacaribe virus (TACV) [2]; however, this virus has not been associated with any disease in humans. Human contact with infected rodents or rodent excrement may result in virus transmission. A multitude of animal models have been used to study the pathogenicity of the arenaviruses and to test product efficacy [3,4]. Most of these models include various rodents comprising of Syrian golden hamsters, mice, and guinea pigs. Though most arenaviruses are rodent-borne viruses, there are no rodent models that completely and accurately mimic the pathogenesis of arenaviral disease in humans. Nonhuman primates (rhesus macaques, cynomolgus macaques, African greens, and common marmosets) have also been used as potential animal models. Experiments utilizing pathogenic hemorrhagic fever-causing arenaviruses such as LASV, JUNV, GTOV, MACV, Chapare virus and SABV all must be performed in a Biosafety Level (BSL)-4 laboratory environment, while other arenavirus research can be conducted in BSL-2 and BSL-3 containment [5]. Small animals provide for certain advantages when working in a BSL-4 laboratory including ease of handling, cost efficiency, and larger numbers of animals allowing for more statistical power. These biosafety containment issues have led to development of surrogate models and various animal models to study arenavirus hemorrhagic fever in a BSL-2/3 environment. 

All of the animal models that are discussed have advantages and disadvantages, but specific aspects of pathogenesis from each model appear to mimic human disease in some capacity. Additionally, pathogenic studies in most of these animal models result in viremia and various levels of lethality, which can be utilized as end points when evaluating prophylaxis and therapeutic efficacy. 

Of the arenavirus-animal model research, the majority of work has focused on Lymphocytic choriomeningitis virus (LCMV) and LASV pathogenesis. LCMV was the first arenavirus discovered during an outbreak of St. Louis encephalitis in 1933 [2,6]. In humans, LCMV has the potential to cause acute aseptic meningoencephalitis of the CNS [3]. LCMV is distributed throughout the world; its natural reservoir, the house mouse (*Mus musculus*) is also distributed throughout the world, which is important since most people infected are likely exposed to infectious rodents [7]. Most infections in humans result in a biphasic mild febrile illness that begins with headaches, fever, malaise, and myalgia [3]. The second phase of disease is marked by neurological signs and may result in meningitis or encephalitis. The mortality rate is less than 1%. By the 1960s, several arenaviruses had been discovered and isolated, including LASV, the etiologic agent that causes Lassa fever [3]. Lassa fever was first described in 1969 in Nigeria during an investigation of a hemorrhagic disease in missionaries [8]. Currently, it is estimated that 100,000 to 300,000 LASV infections occur annually [9], primarily in West Africa. The multimammate mouse (*Mastomys natalensis*) is the reservoir of LASV. Clinical signs of disease may include chest pains, gastrointestinal illness, and pharyngitis [3]. Severe illness occurs in less than 10% of the cases and may include facial edema, hypotension, vasoconstriction, prostrations, and shock. The mortality rate in hospitalized individuals is approximately 15–20% [3]. The duration of the disease is 14–21 days and complications of hearing impairment occur in 29% of known Lassa fever patients [10]. However, the reasons or mechanisms leading to hearing impairment have not been elucidated. Additionally, JUNV, MACV, GTOV, and SABV are the classic four members of the New World arenaviruses (Tacaribe complex viruses) that have the potential to cause viral hemorrhagic fever. Of this group of viruses, JUNV is the most extensively studied. JUNV was discovered in 1957 during a hemorrhagic fever outbreak in Buenos Aires and currently causes an estimated 200 to 2000 cases of Argentine hemorrhagic fever per year [11]. The drylands vesper mouse (*Calomys musculinus*) is the natural reservoir of JUNV [1]. MACV was discovered in 1962 in Bolivia and is the etiologic agent that causes Bolivian hemorrhagic fever; the large vesper mouse (*Calomys callosus*) is the natural reservoir [1]. GTOV is the etiologic agent that causes Venezuelan hemorrhagic fever; a disease which appears to be seasonal [1]. The short-tailed cane mouse (*Zygodontomys brevicauda*) is the natural reservoir. SABV, isolated in 1994, has caused hemorrhagic fever in Brazil [1]. Infection of these viruses in humans may result in a more visible hemorrhagic disease state accompanied with neurologic disease when compared to Lassa fever [1]. Severe disease may include fever, vomiting, headache, malaise, hypotension, petechiae, ecchymoses, hemorrhage, tremors, and seizures. Thrombocytopenia and leukopenia are common clinical pathology findings. The duration of acute illness is normally 10 to 15 days after onset. In severe cases of arenaviral hemorrhagic fever, thrombocyte function may be impaired, which could lead to vascular permeability. However, the exact mechanisms that cause vascular permeability have not been completely characterized. Additionally, it should be stated that mortality can occur with or without vascular leakage, adding further complication to the arenavirus pathogenesis puzzle. In all, this article is designed to review the animal models that have been used to study arenavirus pathogenesis, in addition to linking these animal models to the current prophylactic and therapeutic strategies that are under development. 

## 2. Animal Modeling

### 2.1. The Guinea Pig as a Model for Hemorrhagic Fever

Various strains of guinea pigs, including Strain 13 guinea pigs (inbred) and outbred Hartley guinea pigs, have been used as a model to study Venezuelan hemorrhagic fever [12] and Lassa fever [13] (Table 1). Both strains of guinea pigs develop similar lesions (of various degrees) in the gastrointestinal tract, lung, intestines, vessels, spleen, and lymph nodes after GTOV challenge [12]. Pneumonia has also been reported in both strains of guinea pigs. Additionally, GTOV antigen has been observed in the lungs, liver, lymph nodes, spleen, intestines, lung, heart, brain, and stomach in infected guinea pigs. Hemorrhagic manifestations in the GTOV-infected guinea pig are minimal compared to the manifestations observed in human disease, which includes fever, diarrhea, conjunctivitis, pharyngitis, thrombocytopenia, leucopenia, petechia, epistaxis, melena, and bleeding gums. Infection of guinea pigs with GTOV results in a lethal disease marked by pulmonary and adrenal hemorrhage, bone marrow depletion, and viral antigen in endothelial cells [12]. 

Similarly, LASV pathogenicity has also been studied in Strain 13 guinea pigs and Hartley guinea pigs. Infection of Hartley guinea pigs with LASV results in approximately 30% mortality regardless of the viral dose, whereas Strain 13 guinea pigs are susceptible to lethal infection of LASV (LD_50_ 0.3 PFU) [13]. Peak viremia in Strain 13 guinea pigs occurred 12 days post-challenge. Furthermore, viremia has been observed to develop more rapidly in the Strain 13 guinea pigs when compared to the Hartley guinea pigs, suggesting a higher level of viral replication. Varying degrees of interstitial pneumonia was observed in the moribund animals, in addition to lesions in the kidneys and spleens. However, it should be noted that most lesions were mild. Infectious virus was recovered from all of the extraneural tissues examined (liver, spleen, lymph node, salivary gland, lung, adrenal, kidney, pancreas, heart, and brain) from Strain 13 guinea pigs. 

The pathogenicity of JUNV has also been assessed in both Strain 13 and Hartley guinea pigs, with similar results [14] (Table 1). In this study, the Romero strain caused severe disease signs and complete mortality by 19 days post-challenge in both Strain 13 guinea pigs and Hartley guinea pigs, while the XJ strain caused limited pathogenicity. Additionally, infection of the animals with the JUNV Romero strain caused clinical encephalitis or paralysis, a decrease in body weight, elevated AST levels, thrombocytopenia, and febrile temperatures. In the guinea pig, infection with the Romero strain leads to both a visceral and neurologic form of disease, which is similar to what is observed in humans. Viral titers have been observed in the spleen, liver, adrenal glands, brain, and kidneys and pathologic changes were observed in the liver and spleen. GTOV infection in Strain 13 guinea pigs also leads to complete mortality 11 to 14 days post-challenge [12,15]. Lesions were observed in the gastrointestinal tract and interstitial pneumonia, while viral antigen was associated with the lymphoid tissues, macrophages, endothelial cells of various organs, pulmonary epithelium, and gastrointestinal tract epithelium [12].

Guinea pigs have also been used to study Pichindé virus (PICV) infection as a surrogate model to study LASV fever pathogenesis in a non-BSL-4 environment (Table 1) [16]. Adapted PICV infection in Strain 13 guinea pigs results in mortality, whereas Hartley guinea pigs remain resistant. PICV is not known to be a human pathogen and normally does not affect outbred guinea pigs; however, serial passages in guinea pigs results in an adapted strain that is lethal in Hartley guinea pigs [16,17]. PICV and LASV pathogenesis include a disease progression which culminates in terminal shock in which hemorrhaging is not a major component, high viremia, and virus titers in extraneural tissue. Mortality of guinea pigs after PICV infection normally occurs 13 to 19 days post-challenge. High viremia is associated with the model, in addition to lymphopenia. Lesions (hepatocellular necrosis) are common in the liver of moribund animals, while hemorrhage has been associated with the red pulp of the spleen. The lungs of infected animals normally contained moderate to severe interstitial pneumonia. Because of the disease signs associated with PICV infection in guinea pigs, use of the PICV-guinea pig model appears to be more appropriate as a surrogate to study LASV pathogenesis when compared to the hamster model. Thus, the PICV-guinea pig model system is not only a system that can be used to study arenavirus hemorrhagic fever, but a model that can be used to study drug efficacy against hemorrhagic fever, as long as the drug is not virus specific. 

**Table 1:** Arenavirus—Guinea Pig Models.

Animal	Pathogen	Signs of Disease
Hartley Guinea Pig	GTOV	Lesions in the gastrointestinal tract, lung, intestines, vessels, spleen, and lymph nodes.
		Interstitial pneumonia
		Viral antigen in the lungs, liver, lymph nodes, spleen, intestines, lung, heart, brain, and stomach
		Lethal Disease characterized by pulmonary and adrenal hemorrhage and bone marrow depletion
	LASV	Infection results in 30% mortality and viremia
		Animals not succumbing to disease were relatively resistant to infection
	JUNV	Complete mortality when infected with the Romero strain
		Infection with the Romero strain was associated with encephalitis, paralysis, decrease in body weight, elevated AST, thrombocytopenia, febrile temperatures
		XJ strain caused limited pathogenicity
	FLEV	Lethal in 20% of animals
		Disease marked by weight loss
		No signs of disease in animals that did not succumb to disease
	PICV	Resistant to Infection with adapted PICV strains
		Serial passages of PICV leads to a debilitating viral infection that results in mortality
		Serially passaged PICV infection results in high viremia and viral titers in tissues and culminates in terminal shock
Strain 13 Guinea Pig	GTOV	Lesions in the gastrointestinal tract, lung, intestines, vessels, spleen, and lymph nodes
		Interstitial pneumonia
		Viral antigen in the lungs, liver, lymph nodes, spleen, intestines, lung, heart, brain, and stomach
		Lethal Disease characterized by pulmonary and adrenal hemorrhage and bone marrow depletion
	LASV	Higher lever of viral replication and onset of viremia faster when compared to Hartley guinea pigs
		Interstitial pneumonia
		Lesions in the kidney and spleen
		Uniformed lethality
Strain 13 Guinea Pig	JUNV	Complete mortality when infected with the Romero strain
		Infection with the Romero strain was associated with encephalitis, paralysis, decrease in body weight, elevated AST, thrombocytopenia, febrile temperatures
		XJ strain caused limited pathogenicity
	PICV	Infection with adapted PICV strains results in mortality

### 2.2. The Syrian Golden Hamster as a Model for Hemorrhagic Fever

The Syrian golden hamster is commonly used to study hemorrhagic fever in the PICV surrogate system (Table 2), in addition to testing product efficacy. Infection of hamsters with PICV leads to complete mortality and hemorrhagic fever [18,19]. Infected animals experience significant weight loss, high ALT values, viremia, and viral titers in the liver and spleen. Additionally, an elegant study utilizing Evans blue dye was performed to demonstrate vascular permeability in hamsters infected with PICV [19]. This study marks one of the first to assess changes in vascular permeability in response to hemorrhagic fever virus infection in hamsters. Vascular permeability was associated with Evans blue dye that presumably was leaked into various tissues of PICV infected hamsters. In healthy normal hamsters, the Evans blue dye was contained within the plasma, causing the skin of the animals to change color. Thus, vascular permeability was measured based on the amount of Evans blue dye that leaked in the various tissues. 

A similar vascular permeability study was performed in Syrian golden hamsters infected with the arenavirus Flexal virus (FLEV) [20]. FLEV is a New World arenavirus isolated in Brazil from the *Oryzomys ssp.* (Rice Rat) that has the potential to cause overt disease in humans. In this study, both Hartley guinea pigs (Table 1) and hamsters (Table 2) ranging in age were infected with FLEV. Infection in Hartley guinea pigs only resulted in 20% mortality; no signs of disease were associated with the animals that did not succumb. However, FLEV infection of Syrian golden hamsters (5-6 weeks of age) resulted in 60% mortality; while FLEV infection in hamsters (13–15 weeks of ages) resulted in 80% mortality (mortality was statistically significant when compared to the FLEV-infected guinea pigs). FLEV infection in the hamsters (regardless of age) resulted in hunched posture, ruffled fur, petechiae, hemorrhaging from the mouth, epistaxis, labored breathing, and significant weight loss. Viral titers were associated with the pancreas, kidneys, adrenal glands, heart, lungs, lymph nodes, brain, small intestines, liver, and spleen from terminal hamsters and guinea pigs. Terminal viremia was also measured in all animals that succumbed to disease. No viremia was associated with any of the survivors. Lastly, in similar vascular permeability studies as previously described using Evans blue dye, vascular permeability was associated with specific tissues in all moribund animals, to varying degrees. In all, the results from this study demonstrate that (1) Syrian golden hamsters infected with FLEV may result in mortality that correlates with vascular permeability and (2) provides evidence that this model may serve as an animal model to also study hemorrhagic fever.

**Table 2:** Arenavirus—Syrian Golden Hamster and Mouse Models.

Animal	Pathogen	Signs of Disease
Syrian Golden Hamster	PICV	Infection leads to complete mortality
		Disease is marked by weight loss, elevated AST, viremia, and viral titers in the liver and spleen
		Mortality is associated with vascular permeability
	FLEV	Infection results in 60–80% mortality depending on the age of the animal
		Disease is marked by hunched posture, ruffled fur, petechiae, hemorrhage, weight loss, and terminal viremia
		Viral titers in the pancreas, kidneys, adrenal glands, heart, lungs, lymph nodes, brain, small intestines, liver and spleen
		Mortality may be associated with vascular permeability
	PIRV	Infection results in uniform mortality
		Disease is marked by elevated temperatures, weight loss, viremia, lethargy, petechia, epistaxis, ecchymoses, and neurologic signs of disease
		Hemorrhage observed in the liver, lung, heart, spleen, and brain
MHA Hamster	LCMV	Resistance and susceptibility is dependent on the viral strain
		Infection with LCMV-WE results in viremia, weight loss, and mortality 2–3 weeks post challenge
IFN- α/βγ^-/-^ Mice	JUNV	17%–24% reduction in body weight
		Viral titers in the brain, liver, spleen, and heart
		Lesions in the liver
		Infection leads to mortality
STAT-1 Knockout Mice	MACV	Infection resulting in mortality
		Disease process is dependent on the route of challenge
		Viral titers associated with the spleen, kideny, liver, and lungs
		Increases in ALT and AST levels
		Changes in cytokine and chemokine levels
		Histopathological changes in the liver, thymus, spleen, lymph nodes, and pancreas
CBA Mice	LASV	Intracerebral infection with LASV (Josiah) results in 80–100% mortality
		Clinical signs of disease: weight loss, ruffle fur, loss of mobility, and paralysis
SWR/J mice		Infection results in mild state of disease
		Infection results in severe disease, high viremia, and viral titers in specific tissues

Syrian golden hamsters have also been used as a model system (Table 2) to study hemorrhagic fever associated with Pirital virus (PIRV) infection [21,22,23,24]. PIRV, a New World arenavirus, was originally isolated from the *Sigmodon alstoni* rodent in the Municipality of Guanarito in Venezuela [25]. PIRV has not been associated with human disease and is a BSL-3 pathogen that may serve as a surrogate to study hemorrhagic fever associated with the human pathogenic arenaviruses. PIRV-infected hamsters develop pathology similar to that observed in fatal human cases of arenavirus hemorrhagic fever [24]. Infection of the hamsters with PIRV results in elevated temperatures, loss of body weight, viremia, lethargy, petechia, epistaxis, ecchymoses, and neurologic signs of disease such as tremors, loss of balance, and hind limb weakness or paralysis [21,22]. PIRV infection in hamsters results in complete mortality and post mortem examination demonstrated hemorrhage associated with the liver, lungs, heart, spleen, and brain, splenomegaly, hepatomegaly, and abnormal clinical pathology including elevated ASL and AST levels, as well as an increase in times associated with coagulation. Viral titers are also associated with the lymph nodes, brain, liver, spleen, kidney, heart, intestines, and lungs in terminal animals. Additionally, viremia can be measured 2 days post-challenge and remains constant in animals succumbing to disease. Terminal viremia was also measured in all animals. In all, these data suggest the PIRV-hamster model as a surrogate model to (1) study the disease progression and pathology associated with New World arenavirus hemorrhagic fever and (2) to test vaccine, therapeutic, and/or prophylactic efficacy.

### 2.3. The Mouse as a Model for Hemorrhagic Fever

The mouse model is not as common a model to study arenaviral hemorrhagic fever when compared to the hamster and guinea pig models. However, some investigators have developed murine models to study hemorrhagic fever pathogenesis and treatment efficacy (Table 2) [26,27]. Mice lacking α/β/γ interferon receptors were found to be susceptible to JUNV infection [27]. In this study, IFN- α/βγR^-/-^ mice lost 17% to 24% of their body weight by 13 days post-challenge. Viral loads were associated with the brain, liver, spleen, and heart, with the highest viral loads were found in the kidneys. Histopathology was performed on several tissues and prominent changes were observed in the hearts and brains of these mice in animals euthanized 13 or 14 days post-challenge. Increases in the white pulp volume of the spleens were also observed, while inflammatory infiltrates and necrosis were observed in the liver. Lesions in the liver were also common in JUNV-infected IFN- α/βγR^-/-^ mice. 

STAT-1 knockout mice have been used to study the pathogenesis resulting from MACV infection [26]. These mice succumbed to disease within 7–8 days post-challenge when challenged intraperitoneally with MACV [26]. This route of infection resulted in a rapid and lethal disease when compared to the subcutaneous or intranasal routes of exposure. Viral loads were associated with the spleen and kidney following challenge before disseminating to the liver and lungs. This study also evaluated clinical pathology parameters. An increase in AST and ALT levels were observed 7 days post-challenge, while an increase in white blood cells was observed 5 and 7 days post-challenge. Changes were observed in other clinical pathology parameters, as well. Cytokine and chemokine levels were also evaluated in this study. IFN-γ, IL-5, IL-6, IL-10, MIP-1 α, MIP-1 β, TNF-α, GCSF, and RANTES were all elevated 5 and 7 days post-challenge. MACV challenge also resulted in histopathological changes in the liver, thymus, spleen, lymph nodes, and pancreas. Additionally, this study also tested the efficacy of ribavirin as a post-exposure prophylactic in STAT-1 knockout mice infected with MACV. Ribavirin treatment resulted in 60% survival and a longer time-to-death when compared to untreated mice. Collectively, these data demonstrate that the STAT-1 knockout mice can be used to study MACV-induced pathology and treatment efficacy. 

The mouse has also been used to study LASV-induced pathology. Intracerebral infection of CBA strain mice with LASV (Josiah) (1000 PFU) results in 80–100% lethality 7–9 days post challenge [28]. Clinical signs of disease including weight loss, loss of mobility, ruffled fur, paralysis, and death presented 5 days post-challenge. However, the disease progression associated with this model induces immunopathology more consistent with LCMV disease. In humans and non-human primates, LASV infection does not appear to be related immunopathology. Thus, this model may be useful to evaluate CD8+ T cell involvement, but should not be used to study arenaviral induced disease and product efficacy. 

## 3. The Nonhuman Primate as a Model for Arenavirus Induced Disease

Non-human primates have also been used to study arenavirus hemorrhagic fever (Table 3). The rhesus monkey is an established model to study LASV pathogenesis [29,30,31]. LASV infection in rhesus macaques results in anorexia and febrile temperatures by day 7 post-challenge [32] (Table 3). Clinical signs of disease in LASV-infected rhesus macaques include lethargy, aphagia, constipation, conjunctivitis, and petechia [33]. Viremia can be measured in rhesus 5 days post-challenge and usually peak by day 12–14 post-challenge. Viral titers are associated with the liver, spleen, adrenals, kidney, pancreas, lymph nodes, muscle, heart, thymus, lung, and brain. Microscopic studies have yielded pulmonary, hepatic, renal, and ocular changes with lesions associated with necrotizing hepatitis and interstitial pneumonia [32,33]. LASV infection in the rhesus macaque model yields gross microscopic lesions and abnormal clinical pathology including elevations in AST and ALT, and decreases in fibronectin levels. Studies have yielded evidence that though thrombocyte counts may be reduced after LASV infection; these reductions remain in the normal limits [32]. Though thrombocyte counts remain relatively normal in LASV-challenged rhesus macaques, it appears that a reduction in thrombocyte aggregation occurs after LASV infection [34]. Additionally, some coagulation parameters such as activated partial thromboplastin time (APTT) are consistently abnormal during the terminal phase of disease in LASV-infected rhesus macaques [32]. 

LASV challenge in cynomolgus macaques leads to clinical disease marked by febrile temperatures, dehydration, anorexia, facial edema, epistaxis, and signs of depression [35] (Table 3). Some of the challenged animals have also demonstrated neurologic symptoms including convulsions and seizures and abnormal clinical pathology parameters. As with the rhesus macaque-LASV model, thrombocyte numbers declined but stayed within the normal range in cynomolgus macaques challenged with LASV. Thrombocytopenia is a hallmark of the New World arenaviruses that cause hemorrhagic fever; however, it does not appear to be prominent in humans suffering from Lassa fever. AST and ALT levels increased in LASV-infected-cynomolgus macaques. Additionally, viral titers were detected in the ovaries, brain, heart, lymph nodes, lung, adrenals, kidneys, spleen, and liver and peak viremia occurred 13.5 days post-challenge. Lastly, this study provides evidence that early infection of dendritic cells may have a key role in immunosupression, which is commonly observed in nonhuman primates challenged with LASV. 

**Table 3:** Arenavirus–Nonhuman Primate Models.

Animal	Pathogen	Signs of Disease
Rhesus macaque	LASV	Disease is marked by febrile temperatures, anorexia, lethargy, aphagia, constipation, conjunctivitis, and petechia
		Viral titers associated with the liver, spleen, adrenals, kidney, pancreas, lymph nodes, muscle, heart, thymus, lung, and brain
		Morbidity and mortality (less mortality associated with this model when compared to the cynomolgus model)
	GTOV	Disease associated with lethargy, anorexia, febrile temperatures, and viremia
		Mortality is not associated with infection
	MACV	Hemorrhagic disease characterized by diarrheal, anorexia, dehydration, petechia, coagulation deregulation, thrombocytopenia, and prolonged APTT
		Disease state is generally fatal
	LCMV	Disease is dependent on viral strain, strain of the animal, viral dose, and inoculation route
		Intravenous inoculation results in fever, anorexia, diarrheal, orbital hemorrhage, and shock
		Thrombocytopenia and decreased coagulation function occurs
Cynomolgus macaque	LASV	Disease marked by febrile temperatures, dehydration, anorexia, facial edema, epistaxis, and depression
		Disease marked by febrile temperatures, dehydration, anorexia, facial edema, epistaxis, and depression
		Higher mortality associated with model when compared to the rhesus model
		Increased ALT and AST levels
African green monkeys	MACV	Highly susceptible to MACV infection
		Infection results in fever, anorexia, clinical pathology changes, signs of depression, and death
		Hemorrhage commonly observed in the skin, heart, nasal mucosa, and brain
Common marmoset	LASV	Subcutaneous infection results in weight loss, anorexia, fever elevated AST and ALT, and mortality
		Viral loads in the spleen, adrenals, liver, and lung
		Necropsies of infected marmosets results in lung abnormalities, enlarged liver, and splenomegaly
	LCMV	Infection results in death 7-12 days after the onset of disease signs
		Viral antigen in the liver, lung, spleen, pancreas, kidney, and adrenals

Rhesus macaques infected with MACV, the etiologic agent that causes Bolivian hemorrhagic fever, develop hemorrhagic disease characterized by diarrhea, anorexia, dehydration, petechia, coagulation deregulation, severe thrombocytopenia, prolonged APTT, elevated fibrinogen levels, and gross and microscopic lesions [36,37] (Table 3). This disease state is generally fatal, which is influenced by the dose of virus used to challenge, and provides a useful model to study Bolivian hemorrhagic fever [36]. Rhesus macaques infected with GTOV demonstrate signs of disease including lethargy, anorexia, febrile temperatures, and exhibit viremia (Table 3). However, infected rhesus macaques survive infection (this is different when compared to the other hemorrhagic arenaviruses) and demonstrate high levels of specific neutralizing antibodies in convalescent sera [15]. 

African green monkeys are highly susceptible to infection with MACV and the disease progression is similar to what has been described for the rhesus macaque [38,39]. Infection of the African green monkey with MACV results in fever, anorexia, clinical pathology changes, and signs of depression, in addition to hemorrhages in the skin, heart, nasal mucosa, and brain. Additionally, necrosis can be observed in the liver, adrenal cortex, mouth, esophagus, lymphoid tissue, myocardium, and gastrointestinal mucosa. Infection results in death, and it is also common for animals that die later during the disease progression to demonstrate meningoencephalitis and bronchopneumonia. 

As an alternative to cynomolgus macaques and rhesus macaques, several investigators have used the common marmoset (*Callithrix jacchus*) as a small NHP model (typically weighs between 320 and 450 g) to study arenavirus pathogenesis [40,41,42]. Subcutaneous infection of the marmoset with LASV (Josiah) results in overt disease and death. The animals also exhibited reduced stool production, anorexia, signs of depression, fever, and dramatic weight loss which occurred 10 days post-challenge [41]. Elevated AST and ALT was observed beginning 8 days post-challenge. No significant changes in red or white blood cell counts were observed in infected animals, but a gradual decrease in thrombocytes was also noted over the course of the infection. Viremia was detected 8 days post-challenge and significant viral RNA was associated with the liver, lymphoid tissues, and kidneys. Lung abnormalities, enlarged liver, and splenomegaly were common in necropsied tissues of infected animals [41]. Adrenal necrosis and lymphoid depletion were observed in infected marmosets, characteristics associated with LASV disease in humans. Additionally, the marmoset has been used in studies testing vaccine efficacy [42]. In this study, all LASV-infected marmosets died as a result of disease. Similar to the previous reviewed study, elevated ALT, viremia, and viral loads in the spleen, adrenal, liver, and lung were observed in infected animals. All vaccinated animals survived and the signs of disease were ameliorated. In all, this study validates the use of marmosets as a model to study arenavirus pathology or product efficacy.

## 4. Animal Models for LCMV Infection

As previously mentioned, a majority of work has focused on LCMV pathogenesis when compared to other arenaviruses. LCMV infection leads to a very different type of disease when compared to the pathogenic hemorrhagic fever-causing arenaviruses in humans. LCMV infections in humans result in a biphasic mild febrile illness that can potentially lead to a second phase of disease that is marked by CNS signs and may result in meningitis or encephalitis. 

The mouse model has historically been the dominant animal model to study LCMV, which is due to the ease of manipulating various aspects of the mouse including age and genetics and the ability to study various aspects of the disease including viral persistence, autoimmunity, viral clearance, and viral pathogenesis. In mice, LCMV challenge by a transplacental passage of virus to the fetus from the pregnant mother or by neonatal inoculation, results in a persistent infection. Persistent infection in mice results in high viremia and viral titers in specific tissues. Persistently infected mice also shed virus through their urine for the life of the animal [43,44]. Additionally, LCMV infection in SWR/J mice results in severe disease, while infection in BALB/c mice results in a mild disease state [45], demonstrating the importance of the strains of the mouse that is used.

Both rats and various strains of hamsters have been used to study various aspects of LCMV infection. Rats have been used to study neuropathological aspects of LCMV disease. Infection of young rats (1 to 7 days old) leads to abnormal cerebellar development [46]. LCMV challenge by an intracerbral route leads to permanent cerebellar hypoplasia and ataxia that is nonfatal. This type of infection also leads to virus persistence for up to 4 months following challenge. Like the mouse model, various strains of hamsters are susceptible to LCMV infection, while some strains of hamsters are resistant. Resistance and susceptibility of the hamster depends on the LCMV strain used to inoculate the animals. However, immunosupression of hamsters can lead to LCMV susceptibility [47]. When MHA hamsters are inoculated with the WE strain of LCMV, the animals demonstrated viremia and experience weight loss and death 2 to 3 weeks post-challenge that results from severe wasting [48]. 

LCMV may be used as a surrogate to study hemorrhagic fever in some nonhuman primate animal models, as well. The disease course and type of the disease observed is dependent on various factors including the viral strain, strain of the animal, the administered viral dose, and the route of inoculation. Intravenous inoculation of the rhesus macaque with LCMV leads to clinical signs of disease including febrile temperatures, anorexia, diarrhea, orbital hemorrhage, and shock [49]. Clinical pathology changes were also observed in the animals that were intravenously inoculated. This study also demonstrated that the route of inoculation is of importance to the disease state since the animals inoculated intravenously developed a rapid disease state when compared to the animals that were challenged by gavage. Challenge of animals by gavage did not lead to a disease state as observed with the animals challenged intravenously [49]. The disease associated with animals challenged intravenously closely resembled human and nonhuman primate disease states. LCMV intravenous challenge of rhesus macaques led to thrombocytopenia and decreased coagulation function [49]. In all, this study demonstrates that the inoculation route can influence the disease progression and offers evidence that the intravenous LCMV rhesus macaque model may serve as a good model to study arenaviral hemorrhagic fever and to test vaccine, therapeutic, and prophylactic products. Additionally, a sequential study demonstrated protection in rhesus macaques inoculated with LCMV-ARM and challenged with LCMV-WE [50]. A significant increase in CD3^+^ was observed 2 weeks after infection in the intravenously inoculated animals, which corresponds to the increase observed in CD8^+^ T cells. In contrast, this study demonstrated the opposite pattern in animals inoculated by gavage. These animals exhibited slightly lower CD3^+^ and CD8^+^ cells prior to challenge, but a second challenge resulted in elevated CD8^+^ T cells. This study demonstrated cross protection between the LCMV strains in the monkeys.

Pathological studies involving LCMV and marmosets have also been performed. Common marmosets experimentally infected with LCMV died 7 to 12 days after the onset of disease signs [51]. The liver appears to be the primary target of LCMV; hepatic necrosis in LCMV-infected animals was prominent and tissue injury appeared to be attributed to a direct cytopathic effect. Immunohistochemistry staining of tissues resulted in the biodistribution of LCMV antigens in the liver, lung, spleen, pancreas, kidney, and adrenals of infected animals, which is similar to the biodistribution of LASV antigen in infected rhesus macaques [51]. The results from this study suggest an immune-mediated pathogenesis because a more prolonged course of disease associated with lymphocytic infiltrates as opposed to mortality and acute liver damage usually occurring prior to an antibody response [51].

## 5. Therapeutics and Prophylactics

Therapeutics and prophylactics consist of products that can be administered in the event or likely event of a pathogen exposure. A therapeutic is a compound or product that can be administered to a person at the first signs of disease, while prophylactics are products that can be administered after the possible exposure to a pathogen (post-exposure prophylactic) or prior to the exposure to a potential pathogen (pre-exposure prophylactic). Post-exposure prophylactics (PEP) have been commonly used to treat a variety of pathogen exposures such as Rabies virus (consisting of rabies vaccine and immunoglobulins) and Human immunodeficiency virus (HIV) (AZT). Pre-exposure prophylactics (PrEP) are administered to prevent disease when the possibility of pathogen exposure is high. The various products or potential strategies reviewed in this article can be classified as prophylactics or therapeutics (Table 4).

Immune serum treatment has led to protection in marmosets and guinea pigs infected with JUNV [40,52]. Illness and death were prevented in JUNV-infected guinea pigs treated with immune serum up to 6 days post-challenge; however, some surviving animals developed neurological signs of disease and hind-limb paralysis. Examination of the brains of the treated JUNV-infected guinea pigs revealed viral titers, encephalitis, and meningitis. Serum-treatment of JUNV-infected marmosets led to 75% survival when treated 6 days post-challenge, while all control animals died. All infected animals exhibited anemia, leukopenia, thrombocytopenia, and weight loss. The infected treated animals began dying 21 days post-infection and no deaths were observed after 29 days post-challenge. The control animals all died between days 22 and 32 post-challenge. Hematology values and weights returned to normal in the surviving animals 2 months and 4 months after challenge, respectively. Lastly, immune serum treatment led to the presence of neutralizing antibodies in most of the treated animals (neutralizing antibodies were not detected in one of the treated animals). In all, these studies demonstrate immune serum as a treatment for JUNV induced disease.

As with immune serum, much efficacy research has been performed in arenavirus-infected experimental animals treated with ribavirin. One such study involved using ribavirin as a prophylaxis to treat JUNV-infected rhesus macaques [53]. In this study, ribavirin was administered to the animals at the time of infection of a lethal strain of JUNV (P3790). Ribavirin treatment led to protection from clinical disease in all animals. Ribavirin used in a therapeutic regimen (animals treated at the onset of illness) resulted in an amelioration of disease signs. However, 2 of the 3 animals developed a late central nervous system infection which proved to be fatal. Additionally, ribavirin has been assessed as a treatment of Lassa fever [54]. Human serum AST levels of greater than or equal to 150 resulted in a case-fatality rate of 55 percent [54]. Intravenous ribavirin treatment in human patients, exhibiting similar AST levels within 6 days of the onset of fever, resulted in a statistically significant reduction in the fatality rate (overall mortality was 5%). Ribavirin treatment in patients who presented with a fever for at least 7 days resulted in a 26% fatality rate. It should be noted that oral ribavirin also significantly reduced mortality when evaluating similar risk groups, while LASV-convalescent plasma did not significantly reduce mortality in any of these risk groups. Altogether, these data provide evidence for ribavirin as an effective therapy to treat Lassa fever.

In addition to immune therapy and ribavirin, several experimental therapies have been or are under development. Favipiravir (T-705) (6-fluoro-3-hydrozy-2-pyrazinecarboxamide) is a pyrazine derivative, originally described as an anti-influenza compound [55,56,57], that has shown potential as a therapeutic for arenaviral infections [58]. T-705 potentially targets the RNA polymerases of RNA viruses and was found to be comparable and effective as an antiviral when compared to ribavirin in various arenaviruses in cell-based assays. Administration of the drug to hamsters provided complete protection when infected with PICV. Additionally, studies have been performed with T-705 in guinea pigs infected with an adapted PICV strain [59]. T-705 treatment of PICV-infected animals resulted in animal recovery from disease as evident by reductions in febrile temperatures, viremia, and aspartate aminotransferase (AST), an enzyme that is typically a biomarker for liver health. Furthermore, animals treated with various doses of the drug resulted in increased survival when compared to animals that were treated with a placebo. Moreover, T-705 has been shown to disrupt early/intermediate stages of viral replication [60]. Inhibition of arenaviral infection was demonstrated for a myriad of arenaviruses including the viruses that cause hemorrhagic fever: JUNV, MACV, and GTOV. Therefore, T-705 remains a potential therapeutic to treat arenavirus infections and is likely less toxic than ribavirin, which is likely due to its ability to only weakly inhibit IMP-dehydrogenase and RNA and DNA synthesis when compared to ribavirin. 

A replication-deficient adenovirus type 5 vector (rAd5) that promotes the expression of cIFN-α (DEF201) has also been recently tested as a PrEP and PEP [61]. IFN-α is part of the host innate immune response to viral infections and may provide for a prophylactic for arenaviral infection. cIFN-α has been shown to be effective in hamsters infected with PICV when used alone or in combination with ribavirin [18,62]. These studies demonstrate that treatment of hamsters infected with PICV results in an increase in survival and a reduction in viremia. The combination of ribavirin and cIFN-α resulted in synergistic activity that delayed the progression of disease signs and decreased fatality rates. However, this treatment is hindered due to the short half-life associated with cIFN-α. Thus, the DEF201 was developed and used as a single intranasal administration [61]. DEF201 resulted in a longer enhancement of the host antiviral response and proved to be efficacious as a PrEP and PEP as measured by a significant increase in survival and lower viral titers associated with specific tissues. The pathogenic arenaviruses have evolved strategies to suppress the immune response and these viruses cannot block the induction of exogenous type I IFN, which may be a reason why this strategy proved to be efficacious in this arenaviral model. Additionally, DEF201 appears to be stable, is easy to administer, and can be stored for long periods of time. Because of these factors, DEF201 may prove to be an effective treatment to treat arenaviral disease. 

**Table 4:** Prophylactics and Therapeutics Summary.

Product	Pathogen	Model	Result Summary	Mechanism
T-705	adapted PICV	Guinea Pig	Increased survival in treated animals	Disrupts early/intermediate viral replication
			Reductions in fevers and viremia, and reductions in AST levels.	
DEF201/Ribavirin	PICV	Hamsters	Increased survival and decrease in viremia	Promotion of cIFN-α
TFP	JUNV/TACV	Cell Culture	Reduction in viral replication	Interferes with viral penetration
CPZ	JUNV/TACV	Cell Culture	Reduction in viral replication	Early entry inhibitor
ST-336	TACV	Newborn mice	Increased survival and a delay in mortality	Inhibits viral entry
ST-294	TACV	Newborn mice	Increased survival and a delay in mortality	Inhibits pH-induced membrane fusion
ST-193	LASV	Strain 13 guinea pigs	Less severe disease, lower viremia, and enhanced survival	Inhibits pH-induced membrane fusion
Kinase Inhibitor Cocktail	PIRV	Hamsters	Increased survival and decrease in viremia and viral titers	Inhibits kinases required for viral infection
Immune serum treatment	JUNV	Guinea pigs	Increased survival	Increase of neutralizing antibodies
Ribavirin	JUNV	Rhesus macaques	Protection from clinical disease	Induction of mutation in RNA-dependent replication

Additionally, other compounds like phenotiazine derivatives have been evaluated for their potential replication inhibitory activity. However, it appears that this treatment affects different arenaviruses differently. Both JUNV and TACV replication was susceptible to treatment of cells with the phenotiazine derivatives trifluoperazine (TFP) and chlorpromazine (CPZ) [63], but there appears to be less of an effect when treating cells infected with PICV. Thus, this drug may be more specific in nature to the various arenaviruses and may not be a good candidate as a broad-spectrum arenavirus antiviral. Additionally, only adding CPZ to cells during the early stages of viral replication appears to reduce JUNV, while TFP appears to inhibit replication when present during viral adsorption or when present during replication cycles [63]. The results from this study allowed for the authors to conclude that CPZ was an entry inhibitor, while TFP interfered with the viral penetration process [63]. Brassinosteroids (BRs) are a group of steroidal plant hormones that have a role in growth and differentiation of plant cells. BRs have been shown to inhibit replication of JUNV, TACV, and PICV, as well as other RNA and DNA virus without inactivating the virus [64,65]. Treatment of cells with the synthetic BR 6b ((22S,23S)-3β-bromo-5α,22,23-trihydroxystigmastan-6-one) led to inhibition of viral infection that appeared to mainly affect an early event associated with the viral growth cycle, without inhibiting adsorption or internalization of the virus. The compound seems to affect virus RNA replication by preventing antigenomic RNA synthesis. 

Small molecule inhibitors have also been shown to be effective in inhibiting arenaviral infection. ST-336 and ST-294 have been shown to be efficacious in inhibiting arenaviral infection *in vitro* and in the TACV-newborn mouse model [66]. ST-336 showed antiviral activity in host cells infected with MACV, GTOV, and JUNV by binding to the glycoproteins present on specific arenaviruses and inhibiting viral entry. Additionally, newborn mice treated with ST-294 demonstrated both increased survival and a delay in mortality, suggesting that these small molecule inhibitors may provide an efficacious treatment in humans infected with arenavirus-induced hemorrhagic fever and disease. Further studies demonstrated that ST-294 inhibits pH-induced dissociation of the GPC G1 receptor-binding subunit, a process associated with the viral fusion process [67]. Thus, the compound inhibits infection by inhibiting pH-induced membrane fusion in the endosome, a process critical to productive infection. This study also tested other entry inhibitors, ST-161 and ST-193. It was demonstrated that ST-193 inhibits viral fusion in a similar mechanism when compared to ST-294. The efficacy of ST-193 has also been tested in various *in vitro* and small animal studies [68,69]. Strain 13 guinea pigs were treated with ST-193 prior to LASV infection. Treatment of the guinea pigs with the drug also continued once daily for 14 days. The ST-193 animals exhibited less severe disease and few disease signs. Enhanced survival was observed in the treated animals when compared to the ribavirin and control groups. ST-193 treatment also resulted in lower viremia levels when compared to the control groups. Altogether, these studies suggest small molecule entry inhibitors as a means to treat arenavirus-induced hemorrhagic fever. 

Arenaviruses have been shown to bind to different cellular receptors during viral entry and it appears that different arenaviruses may enter cells through different mechanisms or may utilize multiple entry mechanisms during entry into host cells. For instance, LASV has been shown to bind to the cellular α-dystroglycan, while JUNV, MACV, and GTOV utilize the cellular transferring 1 receptor (TfR 1) [70]. The utilization of different cellular receptors and possible different entry mechanisms presents a challenge to utilizing entry inhibitors to inhibit arenaviral entry into host cells. However, it appears that although these viruses may enter cells through different or multiple mechanisms, arenaviruses appear to require cellular kinase activity for successful cellular entry and productive infection. Thus, it is possible to target the cell processes necessary for entry and productive viral infection as opposed to targeting the specific receptors. Reversible protein phosphorylation events control cell signaling in eukaryotic cells and trigger the “on/off” switch within molecules and/or proteins. Targeting the cellular phosphorylation events required for viral entry may result in the development of therapeutics aimed at inhibiting viral entry and/or infection in host cells, yet more advanced research is needed. Additionally, these data suggest that targeting cellular processes necessary for productive viral infection, as opposed to targeting the virus, may result in a less chance of resistant viral mutants. 

Studies have demonstrated that isoflavones have a collaborative effect on viral entry and cellular signaling [71,72]. Isoflavones are organic compounds found in a variety of plant species and often exhibit antioxidant properties [73]. Some reports suggest isoflavones have the ability to affect virus binding, entry, replication, translation, and formation of viral envelope proteins [71,74]. One of the most extensively studied isoflavones is genistein, a tyrosine kinase inhibitor [71,75,76,77]. Genistein can also block the effects of platelet-derived growth factor and inhibit kinase activity, which can result in the blocking stimulation of c-fos, c-jun, and juB mRNA [78,79,80]. The drug was first discovered in 1899 from *Genista tinctoria* (Dyer’s Broom) [81] and has a chemical composition of 4',5,7-trihydroxyisoflavone. Genistein is the aglycone form of genistin, which results after conversion, *in vivo*, which normally occurs in the stomach by acid hydrolysis. Much of the current research surrounding genistein utilizes direct isolation from soybeans and other plant products as an antioxidant [73]. Genistein has also been observed to reduce arterial contractions in ovariectomized hypertensive rats and to inhibit tyrosine kinase signaling in guinea pigs [82,83]. Since the drug is known to act as a kinase inhibitor, immune modulator, and phytoestrogen [84], this compound may be a valuable candidate that warrants further exploration as a potential prophylactic and/or therapeutic for various viral infections. 

Genistein has been used to deduce the viral entry pathways of a number of viruses [23,84]. Additionally, the drug has been shown to inhibit infection of many different viruses including Herpes simplex virus, Bovine herpes virus type 1, Bovine viral diarrhea virus, Epstein-Barr virus, and Rotavirus [85,86,87,88]. Interestingly, depending on the cell type, either genistin or genistein inhibits productive infection of some viruses. Genistein has been shown to block the up-regulation of c-myc and c-jun induced by Simian virus 40 infection of host cells without inhibiting virus entry [89]. Some arenaviruses like PICV and JUNV have been shown to enter cells by a clathrin-dependent endosomal entry pathway [90,91]. Furthermore, the clathrin-dependent endosomsal entry pathway appears to be mediated through tyrosine kinase activity, and inhibiting tyrosine kinase activity may lead to the inhibition of arenaviral entry and productive infection even though these arenaviruses may enter cells through different endosomal mechanisms. In fact, treatment of cells with the drug Genistein resulted in the inhibition of PICV entry into host cells [71,92], and thus may serve as a model for a potential therapeutic to inhibit arenaviral entry. 

*In vitro* studies utilizing the arenavirus PICV demonstrate an inhibition of viral entry that appears to be mediated by a cholesterol-dependent, non-caveolar, clathrin endocytic pathway that is dependent on endosomal trafficking through dynamin 2, Rab5 and RAb7-mediated endosomes [71,90,92]. These studies identified an endosomal pathway that requires a number of phosphorylation-dependent events for entry and productive infection in host cells. As a result of the necessity for kinase activity in the arenaviral entry pathway, a subsequent study tested the effects of a specific kinase inhibitor as a potential antiviral. The kinase inhibitor genistein was investigated as a potential antiviral against arenaviruses. Pre-treatment of cells with genistein led to an inhibition of PICV infection in host cells [71]. Additionally, genistein pre-treatment resulted in the inhibition of viral transduction in cells that were infected with a LASV-MLV pseudotyped virus that expressed the LASV env and MLV core [71]. However, it should be noted that the pseudotyped system does not completely mimic viral infection and would not cause significant pathology, *in vivo*; the system mainly mimics the viral entry process. Thus, conclusions based on the pseudotyped system should be validated with live wild-type virus (reviewed below). Moreover, the phosphorylation of ATF-2 and CREB (ATF-2 and CREB are two transcription factors that have been implicated in PICV infection), induced by PICV infection were found to be inhibited in cells treated with genistein [71]. These data suggest that the kinases that phosphorylated ATF-2 and CREB may act as targets for the kinase inhibitor genistein.

Furthermore, cells treated with genistein 48 h and 72 h post-PICV infection resulted in the inhibition of virus propagation. These results demonstrate that the kinase inhibitor genistein not only inhibits viral entry, but also inhibits the viral replication cycle post-entry and thus viral propagation. Similar infection inhibition was observed in cells treated with genistein post RV infection [86]. In all, results from these studies suggest that kinase inhibitors like genistein may be tested as a potential prophylactic or therapeutic for VHF in animal models. To test this hypothesis, *in vitro* and *in vivo* studies using the PIRV were performed. PIRV is a New World arenavirus that serves as a surrogate for the Old and New world arenaviruses known to cause hemorrhagic fever in humans [21,22,24]. As previously discussed, infection of hamsters with PIRV produces disease signs that are classically associated with hemorrhagic fever manifestations, including inflammation/lesions in various organs, core temperature increase, weight loss, viremia, petechial rash, hemorrhage, and mortality [22]. Thus, the PIRV-Syrian golden hamster model was utilized to test the affects of the drug, *in vivo* [23]. Animals were separated into groups and treated with genistein (15mg/kg) at several time points pre- and post-challenge. Treating the animals prior to infection, post-pathogen exposure, and at the first signs of disease would allow for the evaluation of efficacy of the drug when administered as a PrEP, PEP, and therapeutic. Animals were challenged intraperitoneally with PIRV and were monitored for signs of disease, which included elevated temperatures, petechial rashes, loss of body weight, lethargy, epistaxis, huddled posture, ecchymoses, ocular and rectal hemorrhage, loss of balance, and hind limb paralysis. Infection of hamsters with PIRV has been shown to result in 100% mortality 6–9 days post-infection [23]. However, PIRV-infected animals treated with genistein as a PrEP, PEP, or therapeutic exhibited fewer signs of disease and increased survival rates. The data from these animal efficacy studies propose genistein operates as a potential treatment for arenaviral hemorrhagic fever.

Once it was determined that genistein could (1) inhibit viral transduction of LASV-pseudotyped virus and (2) inhibit viral infection and ameliorate arenavirus infection in the Syrian golden hamster, a kinase inhibitor cocktail was constructed containing genistein and tyrphostin AG1478 [93]. This study demonstrated that pre-incubation of cells with this kinase inhibitor cocktail resulted in the inhibition of LASV infection. These experiments validated the LASV-pseudotyped virus data that was generated. In this study, inhibition of LASV infection in cells pre-incubated with either genistein or tyrphostin AG1478 was observed. Additionally, treating cells with various concentrations of both kinase inhibitors resulted in synergistic inhibition of viral infection. Though using kinase inhibitors inhibited LASV infection, *in vitro*, animal studies utilizing the kinases inhibitors to treat LASV infection need to be performed to validate both the *in vitro* and surrogate *in vivo* data that has been obtained. Altogether, these data suggest that a kinase inhibiter cocktail may serve as a new class of therapeutics or prophylactics aimed at treating arenavirus infection in humans. 

## 6. Concluding Remarks and Perspectives

Viral hemorrhagic fever is known to affect millions of people around the world. Limited treatment options are currently available. Ongoing outbreaks worldwide of VHF, especially in regions that do not have access to comprehensive medical care, have produced an urgent need for antiviral treatments, which include prophylactics and therapeutics. Various antivirals have proven effective *in vitro *against hemorrhagic fever viruses; however, the high cost and difficulty of efficacy studies within BSL4 containment levels and the lack of proper animal models deter investigators from developing effective therapeutics. Arenaviruses are a diverse group of viruses that can potentially cause hemorrhagic fever in animals and humans. While a vaccine has proven successful for Argentine hemorrhagic fever (JUNV), other vaccine platforms for other arenaviruses have proven ineffective. Additionally, limited prophylactics and therapeutics result in a lack of products to treat arenavirus hemorrhagic fever. Additionally, the use of inconsistent animal models and lack of BSL4 facilities render research aimed to test product efficacy difficult. To overcome these obstacles, researchers have developed various surrogate animal models to test product efficacy in BSL2 and BSL3 environments. Proper efficacy endpoints need to chosen by the investigator to ensure that the model mimics particular aspects of hemorrhagic fever. This will allow for proper evaluation of the various products that are being tested. Using surrogate models is also an effective method to screen various products prior to performing studies within the BSL4. 

It is important to remember that the results from efficacy studies in various models may vary from animal model to animal model and may not translate to effective treatments in humans. The discrepancies of results may be attributed to various factors including differences in the host immune response. The role of the host cellular immunity response may play a role in recovering from arenaviral disease. In humans and some animal models, recovery from LASV infection does not appear to be mediated by an antibody response, but rather from the cellular immune system clearing viremia and virus and enhancing the chances for survival reviewed in [94]. Additionally, high titers of IFN have been demonstrated in sera from patients suffering from Argentine hemorrhagic fever and higher titers in patients that succumb to disease reviewed in [94]. To add further complication, arenavirus infection in animals can shift from an acute phase to a persistent phase, which is marked by a reduction in the production of infective virus in cells that are permanently infected. Although less virus is produced in persistently infected cells, changes occur in these cells that alter the normal function. These are all important factors when evaluating the results from various animal model experiments. Differences in the host immune response and an understanding of the different host and cellular mechanisms that occur during persistent and latent infection are important when evaluating data. In all, the differences in host cell immunity may account for efficacy differences observed with the same product in various animal models.

In conclusion, there have been scientific strides in developing animal models, in order to test prophylactics and therapeutics, and various novel prophylactic and therapeutic products. This has led to the development of products that may potentially combat arenaviral hemorrhagic fever in the future. 
